# Associations between perceived and actual risk of HIV infection and HIV prevention services uptake among men who have sex with men in Shandong province, China: a cross-sectional study

**DOI:** 10.1186/s12889-024-18985-x

**Published:** 2024-06-01

**Authors:** Yuxi Lin, Chuanxi Li, Meizhen Liao, Kedi Jiao, Jing Ma, Yu Yan, Yijun Li, Taoyu Wu, Chunxiao Cheng, Yanwen Cao, Wenwen Jia, Zhonghui Zhao, Lina Wang, Dongdong Hua, Ruixiao Li, Ningning Guo, Jing Meng, Wei Ma

**Affiliations:** 1 Department of Epidemiology, School of Public Health, Cheeloo College of Medicine, Shandong University, Jinan, China; 2 Qilu Hospital of Shandong University, Jinan, China; 3 Institution for AIDS/STD Control and Prevention, Shandong Center for Disease Control and Prevention, Jinan, China

**Keywords:** Perceived HIV risk, Actual HIV risk, HIV testing, Pre-exposure prophylaxis, Post-exposure prophylaxis

## Abstract

**Background:**

Associations between perceived and actual risk of HIV infection and HIV prevention services uptake are inconclusive. This study aimed to evaluate the discrepancy between the perceived and actual HIV risk, and quantify the associations between perceived and actual risk of HIV infection and three HIV prevention services utilization among men who have sex with men (MSM) in Shandong province, China.

**Methods:**

A cross-sectional study was conducted in Shandong province in June 2021. Participants were eligible if they were born biologically male, aged 18 years or older, had negative or unknown HIV status, and had sex with men in the past year. Participants were recruited online. The discrepancy between their perceived and actual risk of HIV infection was evaluated by calculating the Kappa value. Bayesian model averaging was used to assess the associations between perceived and actual risk of HIV infection and HIV prevention services uptake.

**Results:**

A total of 1136 MSM were recruited, most of them were 30 years old or younger (59.9%), single (79.5%), with at least college education level (74.7%). Most participants (97.4%) perceived that they had low risk of HIV infection, and 14.1% were assessed with high actual risk. The discrepancy between their perceived and actual risk of HIV infection was evaluated with a Kappa value of 0.076 (*P* < 0.001). HIV testing uptake had a weak association with perceived high HIV prevalence among social networks (aOR = 1.156, post probability = 0.547). The perceived high HIV prevalence among national MSM was positive related to willingness to use PrEP (aOR = 1.903, post probability = 0.943) and PEP (aOR = 1.737, post probability = 0.829). Perceived personal risk (aOR = 4.486, post probability = 0.994) and perceived HIV prevalence among social networks (aOR = 1.280, post probability = 0.572) were related to history of using PrEP. Perceived personal risk (aOR = 3.144, post probability = 0.952), actual risk (aOR = 1.890, post probability = 0.950), and perceived risk among social networks (aOR = 1.502, post probability = 0.786) were related to history of using PEP.

**Conclusions:**

There is discordance between perceived and actual personal risk of HIV infection among MSM in China. HIV risk assessment and education on HIV prevalence among MSM should be strengthened to assist high-risk populations aware their risk accurately and hence access HIV prevention services proactively.

**Supplementary Information:**

The online version contains supplementary material available at 10.1186/s12889-024-18985-x.

## Background

Men who have sex with men (MSM) are under disproportionate burden of human immunodeficiency virus (HIV) infection. Globally, HIV prevalence was 7.5% among MSM in 2022 [1]. In China, the HIV prevalence among MSM was up to 4.5% in 2022 [2]. To date, several behavioral or biomedical strategies have been recommended to prevent HIV infection, including HIV testing, pre-exposure prophylaxis (PrEP), and post-exposure prophylaxis (PEP) services [3–5]. HIV testing is the crucial first step of HIV prevention and treatment cascade, which could reduce HIV transmission by improving early diagnosis and linkage to care. PrEP and PEP are highly effective biomedical HIV prevention strategies for HIV-uninfected individuals, referring to the use of antiretroviral medication before and after potential HIV exposure to block the acquisition of HIV infection, respectively. Despite great efforts have been taken on the promotion of these HIV prevention services, they are still underutilized among key populations in China and around the world [1, 6, 7]. Globally, about 14% of people living with HIV were unaware of their HIV infection status in 2022 [1], only 50.0% and 58.6% of MSM were aware of and willing to use PrEP [6], and 51.6% and 6.0% of MSM were aware of and ever used PEP respectively [7]. In China, only 61.2% of MSM have tested for HIV and confirmed their HIV status in 2022 [2]. A Chinese meta-analysis estimated that the acceptability of PrEP varied widely across the country in China, ranging from 19.1% to 94.6%, with a pooled estimate of 66.8% (95% CI: 62.0%-71.3%) [8]. A cross-sectional study in China showed that 67.2% of MSM were aware of PEP, 76.0% were willing to use PEP, and 2.3% had ever used PEP [9]. Hence, further research is needed to assess barriers and improve the underutilization of HIV prevention services among eligible MSM.

Actual risk of HIV infection can be evaluated based on individuals’ sexual behaviors. A previous study developed a scale to evaluate the risk of HIV infection among Chinese MSM [10]. Perceived risk of HIV infection is a personal evaluation of how possible a specific person or community would be infected by HIV. Theoretical models of behavior change such as the Health Belief Model [11], Social Cognitive Theory [12], Theory of Planned Behaviour [13, 14], and the AIDS Risk Reduction Model [15] usually assume that risk perception is an essential determinant related to health behaviors. For example, the AIDS Risk Reduction Model proposes that in order to avoid HIV infection, individuals must perceive their sexual behaviors as putting them at risk for HIV infection and then make behavioral changes[15]. However, the relationships between perceived risk and HIV prevention services uptake were controversial. For example, some studies found that self-perceived HIV risk was associated with HIV prevention services, including HIV testing, PrEP and PEP uptake [6, 7, 16, 17]. However, in a German study, risk perception had no association with HIV testing uptake among MSM [18]. Debate also exists in the relationship between actual risk and HIV prevention services uptake. Some studies showed that having risky sexual behavior was positively associated with PrEP willingness and uptake [19, 20], whereas a study in Ghana indicated that there was no significant association between risky sexual behavior and HIV testing uptake [21].

The associations between perceived and actual risk of HIV infection and HIV prevention services uptake are inconclusive. The purpose of this study was to evaluate the discrepancy between perceived and actual HIV risk and quantify the associations between perceived HIV risk, actual HIV risk and HIV prevention services utilization among MSM in China.

## Methods

### Participants

A cross-sectional study was conducted in Shandong province, China in June 2021. Convenience sampling was used to recruit participants by publishing recruitment posters through WeChat (a mobile communication application) and with the help of local community-based organizations (CBOs). Participants were eligible if they were born biologically male, aged 18 years or older, had negative or unknown HIV status, had anal sexual behavior with men in the past year, and provided informed consent.

### Procedures

We developed an online questionnaire on Sojump (an online survey platform) for this study by combining a scale from published literature [10]. The questionnaire was shown in Supplementary file 1. Potential participants could access the questionnaire by scanning the QR code on the recruitment poster and then complete it online. The beginning of the questionnaire consists of five questions about the inclusion criteria and informed consent. Participants who met the inclusion criteria could enter the remaining of the questionnaire. Participants who completed questionnaires were compensated 50 CNY (about 8 USD) for their time and participation. Ethical approval for this study was obtained from the Institutional Review Board at the School of Public Health, Shandong University.

### Measurements

#### Social-demographics

Participants were asked about their age, occupation, education degree, monthly income, marital status with women, gender identity, and sexual orientation. Participants also reported whether they had disclosed their sexual behaviors with men to others (except for their sexual partners) or to doctors.

#### HIV-related knowledge

HIV related knowledge was assessed by eight questions which were widely used by centres for disease control and prevention to evaluate the level of HIV knowledge among high-risk populations. Participants received one point for each question they answered correctly, and the participant’s HIV knowledge score was the sum of these eight questions. Participants who scored six and above were classified as “high HIV-related knowledge”, while those who scored less than six were classified as “low HIV-related knowledge”.

#### Social media use

We asked participants whether they ever used MSM-oriented social applications (e.g., Blued) and whether they browsed information about HIV prevention through mass media (e.g., WeChat, microblog, and QQ). Participants were also asked where they found their sexual partners and if they had ever sought for sexual partners through the web.

#### Perceived risk of HIV infection

This study assessed each participant’s perceived risk of HIV infection through three questions. First, participants were asked to assess their likelihood of being infected with HIV in the past six months (“high possibility”, “low possibility”, or “no possibility”). Those who responded “high possibility” were categorized as “high risk perception of HIV infection”, and those who responded “low possibility” and “no possibility” were categorized as “low risk perception of HIV infection”. In addition, we also asked them about their estimates of the severity of HIV prevalence among MSM population in their social networks and in China (“serious”, “relatively high”, “moderate”, “relatively low”, or “rarely zero”). Those responded “serious”, “relatively high”, or “moderate” were categorized as “high risk perception of HIV infection”, and those responded “relatively low” and “rarely zero” were categorized as “low risk perception of HIV infection”.

#### Actual risk of HIV infection

The actual risk of being infected with HIV was assessed using an HIV risk assessment scale, which was developed specifically for Chinese MSM with high reliability and validity [10]. This scale consists of eight items, and participants received a total score equal to the sum of the scores on all items, ranging from zero to 15. Participants with scores less than five were classified as having “low actual risk of HIV infection” and those with five and higher scores were classified as having “high risk of actual HIV infection”. The items of HIV risk assessment scale were shown in Supplementary file 2.

#### HIV prevention services utilization

We measured the HIV prevention services utilization among participants, including HIV testing uptake, willingness to use PrEP or PEP, and history of using PrEP or PEP. Specifically, participants were asked whether they had tested for HIV in the past six months in healthcare facilities or on their own. Then, after given a detailed introduction about PrEP or PEP services, participants were asked if they had heard of PrEP or PEP before (“Yes” or “No”), whether they would be willing to use PrEP or PEP before and after potential HIV exposure henceforth, respectively (“Yes” or “No”), and whether they had ever used PrEP or PEP before (“Yes” or “No”).

### Statistical analysis

The sample size was calculated based on the sample size calculation formula for cross-sectional study as $\text{n}=\frac{{Z}_{1-\frac{\alpha }{2}}^{2}\times pq}{{d}^{2}}$. The primary outcomes of this study were HIV testing uptake and willingness to use PrEP/PEP among MSM in China. According to previous studies [2, 8, 9], the proportion of having HIV testing and willingness to use PrEP/PEP were 60% and 70% among MSM in China, respectively. Therefore, the sample size was estimated as 267 and 171 MSM with two-sided α set to 0.05 and d set to 0.1 × p. In summary, this study should recruit at least 267 MSM.

Descriptive statistics were used to assess participants’ demographic characteristics, social media use, HIV-related knowledge, perceived and actual risk of HIV infection, as well as HIV prevention services uptake. Kappa value was calculated to assess the discrepancy between their perceived and actual risk of HIV infection. To assess the association between perceived risk, actual risk of HIV infection and HIV prevention services uptake, Bayesian model averaging was used to identify the potential determinants of HIV prevention services uptake. Posterior mean and posterior probability were calculated to reflect the association strength using “BAS” package in R software. Bayesian model averaging could obtain averaged estimates over a range of candidate models selected with equivalent prediction, which could capture model uncertainty within model selection potentially mitigate this issue by averaging across a set of models, with the theory being that the true model is a part of this set [22, 23]. When interpreting the posterior probability, a cutoff of 0.5 is generally used to determine which regressors have evidence of an effect. A value between 0.5 and 0.75, between 0.75 and 0.95, between 0.95 and 0.99, and more than 0.99 indicate weak, positive, strong, and very strong evidence for an association [24].

## Results

### Demographic characteristics of participants

A total of 1136 MSM were recruited, most of them were 30 years old or younger (59.9%, 680/1136), single (79.5%, 903/1136), with at least college education level (74.7%, 849/1136), monthly income of more than 3000 CNY (74.1%, 843/1136), self-identifying as male (96.2%, 1093/1136) and homosexual (59.6%, 859/1136). In terms of HIV prevention services utilization, 82.6% of participants (938/1136) had been tested for HIV in the past six months. About three-quarters of participants had heard of PrEP or PEP before, more than 90% of participants were willing to use PrEP or PEP in case they face incident HIV exposure, and less than 10% had used PrEP or PEP before (Table 1).

**Table 1:** Demographic characteristics of participants (*N* = 1136)

Characteristics	n(%)	Characteristics	n(%)
Age		Ever received HIV-related information through social media	
< 30 years old	680 (59.9)	No	109 (9.6)
≥ 30 years old	456 (40.1)	Yes	1027 (90.4)
Marital status		Ever sought sexual partners through web	
Single	903 (79.5)	No	68 (6.0)
Married	156 (13.7)	Yes	1068 (94.0)
Divorced	77 (6.8)	Participated in HIV/AIDS related activities organized by CBOs	
Highest education level		No	650 (57.2)
High school or below	287 (25.3)	Yes	486 (42.8)
College or beyond	849 (74.7)	Tested for HIV in the past six months	
Monthly income		No	198 (17.4)
< 1500 CNY	103 (9.1)	Yes	938 (82.6)
1500–3000 CNY	190 (16.7)	Awareness of PrEP	
3001–5000 CNY	397 (34.9)	No	291 (25.6)
5001–8000 CNY	314 (27.6)	Yes	845 (74.4)
> 8000 CNY	132 (11.6)	Willingness to use PrEP	
Gender identity		No	45 (4.0)
Male	1093 (96.2)	Yes	1091 (96.0)
Other	43 (3.8)	Ever used PrEP	
Sexual orientation		No	1040 (91.5)
Homosexuality	859 (75.6)	Yes	96 (8.5)
Other	277 (24.4)	Awareness of PEP	
Disclosure of sexual intercourse with men to others		No	273 (24.0)
No	392 (34.5)	Yes	863 (76.0)
Yes	744 (65.5)	Willingness to use PEP	
Disclosure sexual intercourse with men to doctors		No	34 (3.0)
No	668 (58.8)	Yes	1102 (97.0)
Yes	468 (41.2)	Ever used PEP	
Knowledge about HIV		No	1041 (91.6)
Not known	108 (9.5)	Yes	95 (8.4)
Known	1028 (90.5)		
Ever used MSM social software			
No	53 (4.7)		
Yes	1083 (95.3)		

1 CNY=0.155 USD in 2021

### Perceived and actual risk of HIV infection

Table 2 presents the perceived and actual risk of HIV infection among participants. Most participants (97.4%, 1107/1136) perceived that they had low risk of HIV infection. About half of the participants (45.3%, 515/1136) believed that the HIV prevalence among MSM population in their social networks was high, and 87.1% (990/1136) of participants perceived that the HIV prevalence among Chinese MSM was high. Supplementary file 3 shows the details. In terms of actual risk of HIV infection evaluated using HIV risk assessment scale, the majority of participants (85.9%, 976/1136) were assessed as low risk, while 14.1% (160/1136) were assessed as high risk. The scales of each item are shown in Supplementary file 4. Specifically, 44.5% of the participants (506/1136) had multiple sexual partners and 28.7% (326/1136) had unprotected homosexual anal sex in the past six months. Most of them did not have commercial sex with men (96.0%), were not diagnosed with sexually transmitted diseases (98.1%), did not use recreational drugs (86.7%), and did not have group sex with men (86.8%). About twenty percent (17.5%) of the participants were unaware of their sexual partners’ HIV status. As for the sexual roles played in sexual behavior, 29.3% only played insertive role, 36.1% only played receptive role, and 34.6% played versatile role.

**Table 2:** Perceived and actual risk of HIV infection among participants (*N* = 1136)

Risk of HIV infection	n(%)
Perceived risk of HIV infection of themselves	
Low	1107(97.4)
High	29(2.6)
Perceived HIV prevalence among local MSM population	
Low	621(54.7)
High	515(45.3)
Perceived HIV prevalence among Chinese MSM population	
Low	146(12.9)
High	990(87.1)
Actual risk of HIV infection	
Low	976(85.9)
High	160(14.1)

### Discrepancy between perceived and actual risk of HIV infection

Specifically, among 160 participants who were objectively scored as high risk, only 6.9% (11/160) perceived high risk of HIV infection of themselves, 93.1% (149/160) perceived low risk. Among 976 participants were objectively scored as low risk, 98.2% (958/976) perceived low HIV risk, and 1.8% (18/976) perceived high HIV risk. It is noteworthy that among those who had low perceived risk of HIV infection, 13.5% (149/1107) were actually at high risk. The consistency between perceived risk and actual risk was poor (Kappa = 0.076, *P* < 0.001, Table 3).

**Table 3:** Discrepancy between perceived and actual risk of HIV infection

Perceived risk of HIV infection	Actual risk of HIV infection		Kappa	*P* value
	Low	High		
Low	958	149	0.076	< 0.001
High	18	11		

### Relationships between risk of HIV infection and HIV prevention services uptake

**Fig. 1:**
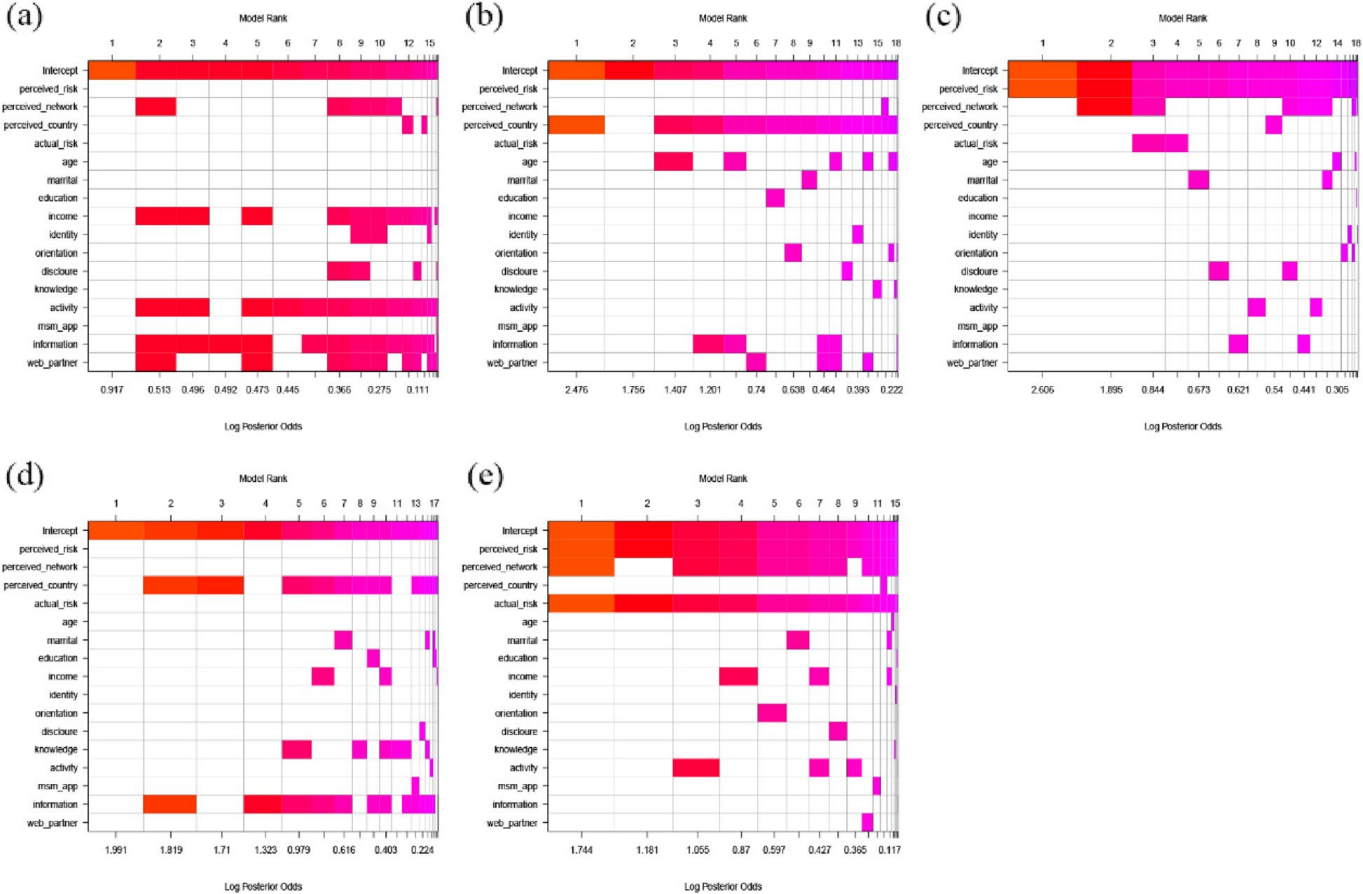
The relationships between actual risk and perceived risk and HIV prevention services. **a** The relationships between actual risk and perceived risk and HIV testing uptake. **b** The relationships between actual risk and perceived risk and willingness to use PrEP. **c** The relationships between actual risk and perceived risk and history of using PrEP. **d** The relationships between actual risk and perceived risk and willingness to use PEP. **e** The relationships between actual risk and perceived risk and history of using PEP. The models are sorted from best on the left to worst on the right based on a posterior probability. The best model is on the left, and the worst model is on the right. The shade of each square represents the posterior probability of each variable. The color of each column is related to the logarithm of the posterior probability (X-axis) of the model, white indicates variables excluded from models

Figure 1a and Supplementary file 5 show the relationship between risk of HIV infection and HIV testing uptake. Perceived high HIV prevalence among social networks (aOR = 1.156, post probability = 0.547) had weak association with HIV testing uptake. It is found that perceived HIV prevalence among national MSM, perceived personal risk and actual risk have no relationship with HIV testing uptake (post probability < 0.5). In addition, monthly income (aOR = 1.198, post probability = 0.718), participating activities conducted by CBOs (aOR = 1.281, post probability = 0.856), contacting HIV-related information through apps (aOR = 1.364, post probability = 0.778), and seeking sexual partner through web (aOR = 1.306, post probability = 0.630) were related to HIV testing uptake.

Figure 1b and Supplementary file 5 show the relationships between actual and perceived risk of HIV infection and willingness to use PrEP. The perceived high HIV prevalence among national MSM (aOR = 1.903, post probability = 0.943) had a strong association with willingness to use PrEP, but the perceived HIV prevalence among social networks and their perceived and actual HIV risk were not related to willingness to use PrEP (post probability < 0.05). Besides, age (aOR = 0.816, post probability = 0.516) had a weak negative association with willingness to use PrEP. Figure 1c and Supplementary file 5 show the relationships between actual and perceived risk of HIV infection and history of using PrEP. Perceived personal risk had a very strong association with history of using PrEP (aOR = 4.486, post probability = 0.994). Perceived HIV prevalence among MSM in their social networks had a weak association with history of using PrEP (aOR = 1.280, post probability = 0.572). The perceived risk of MSM in China and actual HIV risk were not related to history of using PrEP (post probability < 0.5).

Figure 1d and Supplementary file 5 show the relationships between actual and perceived risk of HIV infection with willingness to use PEP. The perceived HIV prevalence among national MSM had a positive association with willingness to use PEP (aOR = 1.737, post probability = 0.829). Perceived HIV prevalence among social networks, perceived personal HIV risk and actual HIV risk had no relationship with willingness to use PEP (post probability < 0.5). Contacting HIV-related information through apps was related to willingness to use PEP (aOR = 1.893, post probability = 0.772). Figure 1e and Supplementary file 5 show the relationships between actual and perceived risk of HIV infection with history of using PEP. Perceived personal risk (aOR = 3.144, post probability = 0.952), actual risk (aOR = 1.890, post probability = 0.950), perceived risk among their MSM social networks (aOR = 1.502, post probability = 0.786) had strong association with history of using PEP. The perceived risk of national MSM were not found to be related to the history of using PEP (post probability < 0.5).

## Discussion

We conducted a cross-sectional survey among MSM in Shandong province, China to evaluate the discrepancy between perceived and actual HIV risk, and assess the relationships between actual and perceived risk of HIV infection and three main HIV prevention services. This study found a discrepancy between perceived and actual risk of HIV infection, and a bias that participants perceived lower HIV prevalence among people who were more proximal to them than distal counterparts. HIV testing uptake had a weak association with perceived HIV prevalence among MSM in their social networks. The willingness to utilize PrEP or PEP were related to the perceived HIV prevalence in China, but the history of using PrEP or PEP were related to perceived personal risk of HIV infection. It is imperative to strengthen HIV risk assessment among MSM to help them understand their risk accurately and further proactively seek HIV prevention services. Besides, this study also underlined the necessary of improving knowledge of HIV epidemic among MSM in their social networks and in China to improve the willingness of using HIV prevention services.

In this study, most MSM perceived that they had low risk of acquiring HIV, while perceived the HIV prevalence among MSM in their social networks and in China were high. This finding was similar to a previous study among MSM in the United States, which found that participants believed that the prevalence among MSM was 21% nationwide, 20% in their home state, 7% among their MSM friends and 6% among their male sex partners, but 28% reported high-risk sexual behaviors and only 3.6% perceived their risk of HIV infection [17]. Another study among MSM in United States also reported that MSM perceived the higher HIV prevalence among MSM in country and city than those in their social and sexual networks [25]. The phenomenon that MSM perceived who are closer have a lower HIV prevalence than those who are farther away can be explained by comparative optimistic bias [26]. People were generally unbiased about hereditary risk factors and even somewhat pessimistic about environmental risk factors, but their views of their own actions and psychological attributes were excessively optimistic [27]. Previous studies have demonstrated that how accurately MSM understand their HIV environment may contribute to prevention decisions [17, 25]. In this study, the higher optimism bias might make high-risk people underestimate their risk and miss the best chance to seek HIV prevention services. It was suggested that targeted education and HIV risk assessment should be reinforced to make people aware the accurate HIV risk of themselves, around them, and across the country, as well as make high-risk population seek prevention services early.

It was worth noting that perceived personal HIV risk was discordant with actual risk among MSM in this study. This study found that 97.4% MSM perceived that they had low risk of HIV infection, but of whom 14.1% actually had high risk of HIV infection. However, more than 10% participants with low perceived risk of HIV infection were actually at high risk. This finding was consistent with a study among MSM and transgender women in Thailand, which indicated that among participants who perceived themselves as having low HIV risk, but over 80% reported having risky sexual behaviors [28]. Another study conducted in Uganda also found the inconsistence between perceived and objectively measured risk of HIV infection [29]. The underestimation of HIV infection risk among high-risk population may hinder the HIV prevention services uptake and increase high-risk sexual behaviors [17]. It is suggested to strengthen HIV risk assessment and counselling services to help HIV vulnerable populations objectively evaluate their risk of HIV infection, reduce high-risk behaviors, and utilize HIV prevention services effectively.

The relationships between perceived and actual risk of HIV infection and HIV prevention services varied from different services. The utilization of HIV testing had a weak association with perception of HIV prevalence among social networks, but had no association with actual HIV risk, perceived personal risk, and perceived national HIV prevalence. This finding is similar to a previous study, which found that perceiving higher prevalence among their social and sexual networks was associated with higher odds of lifetime and routine HIV testing among MSM in the United States, but perceiving higher prevalence in the city and country had no significant association [25]. This finding indicates that HIV testing behavior was influenced by multiple factors and innovative interventions should be developed to address barriers hindering people seeking test services. For example, this study found that participating in activities conducted by CBOs and receiving HIV-related information through social media were positively associated with HIV testing uptake. To date, the CBOs have played an essential role in addressing determinants of health through their formal and informal medical and social services [30, 31], and social media have been widely used in HIV prevention programs [32, 33]. More programs including education on HIV prevalence and HIV risk assessment with consulting service could be conducted by CBOs through activities or through social media to make MSM understanding the HIV risk better and seek testing services routinely.

This study found that more than 90% participants reported that they would use PrEP and PEP if they exposed to HIV. The willingness to use PrEP or PEP are both higher in this study when compared to a previous study [6, 34, 35]. A meta-analysis estimated that the willingness to use PrEP ranged from 5.7% to 100%, and the pooled estimate was 58.6% around the world [6]. A previous study conducted among MSM in China found that 70% of participants would be willing to use PEP if exposed to HIV [35]. However, only about 10% of participants with actual high risk of HIV infection had used PrEP or PEP. These results indicate that the high willingness to use prevention services does not transfer to behaviors among high-risk populations. A previous study in China also found that the prevalence of willingness to use PrEP was 45.2% if free PrEP was provided, but only 1% had ever used PrEP [36]. In the present study, the willingness to use PrEP and PEP were related to the perceived HIV prevalence among national MSM, but the history of using PrEP and PEP were predicted by perceived high HIV risk of themselves. This finding, to our knowledge, firstly discovered the priority role of perceived risk of HIV infection in PrEP and PEP utilization. It was suggested that only when high-risk population perceived their risk, can willingness to use HIV prevention services transfer to behaviors of using services, and HIV prevention services promotion programs be implied effectively among high-risk populations. Therefore, except for education on the knowledge of PrEP and PEP services, the HIV risk assessment should be paid more attention to motive population with high risk to seek HIV prevention services actively.

This study has some advantages. Most previous studies explored the relationship between personal HIV risk and a specific HIV prevention service [19, 37, 38], while we quantified the associations between perceived and actual risk and three main HIV prevention services, including HIV testing, PrEP and PEP services. At the same time, this study evaluated the perception of HIV risk from three perspectives, including personal risk, risk among MSM in their social networks, and risk among MSM in China. Therefore, this study could provide generalized and comprehensive guidance for HIV prevention services promotion in high-risk population. In addition, the accuracy of the actual HIV risk among participants was assessed using a HIV risk assessment scale targeting Chinese MSM, as its good performance has been validated [10]. However, some limitations should be acknowledged. First, as a cross-sectional design, we cannot verify the causal relationship since the temporality was unclear. Further cohort study should be conducted to test the casual relationships between HIV risk and prevention services uptake. Second, due to using the relative severity of HIV prevalence to measure the perception of HIV risk among participants’ social networks and in China and the uncertainty of the HIV prevalence among participants’ social networks, the accuracy of participants’ perceptions of the HIV prevalence in their networks and in China is unable to evaluate. Further study should be conducted to evaluate the accuracy of the perceptions of HIV risk in their social networks and in China. Third, the participants in this study were recruited through social media and CBOs, who were not representative of the whole MSM population, as MSM who did not engage with social media and CBOs may be difficult to recruit. Forth, condom use is a concise and effective service to prevent HIV infection, however, this study did not evaluate the relationship between HIV infection risk and condom use since that the actual risk of HIV infection was partly assessed through condom usage, and it is difficult to assess the relationship unbiased.

## Conclusions

There is discordance between perceived and actual personal risk of HIV infection and a bias that HIV prevalence was perceived lower among people who were more proximal to them than distal counterparts among MSM in China. HIV risk assessment among MSM should be strengthened to assist high-risk populations aware their risk accurately and hence access HIV prevention services proactively. In addition, education on HIV prevalence among MSM should also be strengthened to make high-risk populations aware HIV risk around them accurately.

### Supplementary Information

Supplementary Material 1.

Supplementary Material 2.

Supplementary Material 3.

Supplementary Material 4.

Supplementary Material 5.

## Data Availability

Individual participants’ data that underlie the results reported in this article and a data dictionary defining each field in the set are available to investigators whose proposed use of the data has been approved by an independent review committee for work. Proposals should be directed to weima@sdu.edu.cn to gain access, data requestors will need to sign a data access agreement. Such requests are decided on a case by case basis.

## Abbreviations

MSM: Men who have sex with men
HIV: Human Immunodeficiency Virus
PrEP: Pre-exposure prophylaxis
PEP: Post-exposure prophylaxis
CBO: Community-based organization
