# Supplementary material for: Associations between perceived and actual risk of HIV infection and HIV prevention services uptake among men who have sex with men in Shandong province, China: a cross-sectional study
Source: BMC Public Health. 2024 Jun 1;24:1470. doi: 10.1186/s12889-024-18985-x (PMC11143659; doi:10.1186/s12889-024-18985-x)
Supplement: Supplementary file 1 — Supplementary Material 1. [file 12889_2024_18985_MOESM1_ESM.docx]

Supplementary file 1. Online questionnaire (English version).

**Part I**

**Health survey of Shandong University**

**About this Study:**

Hello, we are the research team of Shandong University, thank you very much for participating in our survey!

This questionnaire has many pages, but most of the pages have only 1-2 single-choice questions. Your part in this current survey will last approximately 8-10 minutes. We will send you a red envelope of 50 RMB after you submit the questionnaire.

**Online Consent Form**

**Title of Study:** Health survey of Shandong University

**IRB study number:**20190210
**Principal Investigator:** Dr. Wei Ma
Dr. Wei Ma, PHD tutor, School of Public Health, Shandong University, 44 West Wenhua Road, Jinan, Shandong Province, 250012, China. E-mail: [weima@sdu.edu.cn](mailto:weima@sdu.edu.cn).

**What are some general things you should know about this research studies?** You are being asked to participate in a research study. To join this research study is voluntary. You may for whatever reason refuse to join or withdraw your consent to be in the study at any time. Details about this study are discussed below. It is important that you understand this information so that you can make an informed choice about joining this research study.

**What is the purpose of this study?** This study aimed to access the actual risk and perceived risk of HIV infection among men who have sex with men, and help them to correctly understand their risk of HIV infection, and promote HIV prevention services utilization.

**How many people will take part in this study?** If you decide to participate in this research study, you will be one of approximately 1000 individuals.

**What will happen if you take part in the study?** Your part in this current survey will last approximately 8-10 minutes. During this current survey, you will be asked to complete an online questionnaire. The study questionnaires will ask you to provide sociodemographic information as well as details about your sexual health and sexual activity. Upon completion of this initial questionnaire, you will be asked to input your mobile phone number as a means for the research team to prevent duplicate responses, to send reminders, and to distribute rewards for participation. Your information will be kept strictly confidential. Your mobile and WeChat number will be strictly confidential and will not be used for any other purposes.

**What are the possible benefits from being in this study?** This study aimed to utilize new knowledge to benefit men who have sex with men. The research on HIV risk assessment among men who have sex with men is still in the exploratory stage. The results of this study will help us to understand the actual risk and perceived risk of HIV infection among men who have sex with men in China. Your participation will promote men who have sex with men to correctly understand their own risk of HIV infection, and provide a basis for further targeted education and programs.

**How will your privacy be protected?** All data is directly entered into computers as participants complete the questionnaires. Access to the data will be password protected within the server’s firewall. Cookies will not be used in any way to track participant activity. We will delete all your privacy information like phone and WeChat number immediately after you finish all four follow-up surveys.

**Will you receive anything for being in this study? Will it cost anything? Participants will have the opportunity to earn 50 RMB WeChat red pocket** There are no costs associated with participating in this research study.

**What if you have questions about your rights as a research participant?** All research on human volunteers is reviewed by a committee that works to protect your rights and welfare.

Q1. If you understand and agree to participate in this research study, please select “Agree” from the options below. We thank you for your participation!

○ Agree

○ Decline (Skip to end of survey when finished Q5)

Q2. How would you describe your assigned sex at birth?

○ Male

○ Female (Not eligible to take this survey – Skip to end of survey when finished Q5)

Q3. Please select your age:

○ <18 (Not eligible to take this survey – Skip to end of survey when finished Q5)

○ 18

○ 19

○ 20

○ 21

○ 22

○ 23

○ 24

○ 25

○ 26

○ 27

○ 28

○ 29

○ 30

○ 31

○ 32

○ 33

○ 34

○ 35

○ 36

○ 37

○ 38

○ 39

○ 40

○ 41

○ 42

○ 43

○ 44

○ 45

○ 46

○ 47

○ 48

○ 49

○ 50

○ 51

○ 52

○ 53

○ 54

○ 55

○ 56

○ 57

○ 58

○ 59

○ 60

○ 61

○ 62

○ 63

○ 64

○ 65

○ 66

○ 67

○ 68

○ 69

○ 70

○ >70

Q4. In the last year, have you ever had oral or anal sex with another man?

○ Yes

○ No (Not eligible to take this survey – Skip to end of survey when finished Q5)

Q5. Have you been infected with human immunodeficiency virus (HIV)?

○ Negative HIV testing result/I have not been infected with HIV

○ Positive HIV testing result/I have been infected with HIV (Not eligible to take this survey – Skip to end of survey)

○ I don't know my HIV infection status

**Part II**

**Sociodemographic information**

Q1. Do you live in one of the following cities, and do not have moving plans in the next 1 year?

○ Jinan, Shandong

○ Qingdao, Shandong

○ Weifang, Shandong

○ Zibo, Shandong

○ Jining, Shandong

○ Liaocheng, Shandong

○ Dezhou, Shandong

○ Weihai, Shandong

○ Binzhou, Shandong

○ Heze, Shandong

○ Zaozhuang, Shandong

○ Dongying, Shandong

○ Yantai, Shandong

○ Taian, Shandong

○ Rizhao, Shandong

○ Linyi, Shandong

○ City outside Shandong Province: ____________ (Not eligible to take this survey – Skip to End of Survey)

Q2. Please enter your phone number: _____________

Q3. Which kind of hukou are you currently holding?

○ Current city’s hukou

○ Other urban hukou in the province

○ Rural hukou in the province

○ Urban hukou outside the province

○ Rural hukou outside the province

Q4. How long have you been living in this city? _____ year(s) _____ month(s)

Q5. What is your current residential status?

○ Living alone

○ Living with spouse/girlfriend

○ Living with boyfriend

○ Living with parents/siblings/relatives

○ Living with friends/colleagues

○ Living in collective dormitories

○ Other

Q6. What’s your occupation?

○ Student

○ Employee of government or public institutions (such as civil servant, teacher, doctor, lawyer)

○ Business and service industry personnel (such as chef, waiter, driver)

○ Company employee (white-collar worker)

○ Ordinary worker (blue collar)

○ Farmer

○ Self-employed

○ Sex worker

○ Unemployed

○ Other: __________

Q7. What is your current marital status (referring to woman)?

○ Not married

○ Engaged or married

○ Separated or divorced

○ Widowed

Q8. Do you have any child now?

○ Yes

○ No

Q9. What is the highest education level you have completed?

○ High school or below (including Zhongzhuan)

○ Some college (Dazhuan)

○ College/Bachelors

○ Masters/PhD or above

Q10. What is your monthly income?

○ Less than 1 500 RMB

○ 1 500-3 000 RMB

○ 3 001-5 000 RMB

○ 5 001-8 000 RMB

○ More than 8 000 RMB

Q11. How much is your monthly living allowance?

○ Less than 1 500 RMB

○ 1 500-3 000 RMB

○ 3 001-5 000 RMB

○ 5 001-8 000 RMB

○ More than 8 000 RMB

Q12. What is your gender identity?

○ Male

○ Female

○ Transgender

○ Uncertain/Other

Q13. What is your sexual orientation?

○ Homosexuality

○ Bisexuality

○ Heterosexuality

○ Uncertain/Other

Q14. Have you ever told anyone about your sexuality or sexual history with men (except your sexual partner)?

○ Yes

○ No (Skip to Q16)

Q15. Have you ever told health-care providers about your sexuality or sexual history with men?

○ Yes

○ No

**MSM basic situation**

*The next set of questions will ask you about your sexual behaviors with other men.*
Section 1: Stable male sex partners

*Note: Stable male partners mean male sex partners who maintain a sexual relationship with you more than 3 months (>3 months), including boyfriends and/or regular male sex partners.*

Q16. In your lifetime, have you had any stable male sex partners?

○ Yes

○ No (Skip to Q23)

Q17. How old were you when you had anal intercourse with your stable male sex partner for the first time?

________years old *(Number input)*

Q18. The first time you had sex with your stable male sex partner, was it consensual?

○ Yes

○ No

*Note: Our definition of consensual is that you were interested in having sex with him, but that you felt like you could have refused or stopped it if you wanted to.*

Q19. Where did you mainly meet with your stable male sexual partner(s)? (Select all that apply)

○ Pub, disco, tearoom, or club

○ Spa or bath house, sauna, foot or body massage parlor

○ Park, public restroom, public lawn

○ Website

○ Social media

○ Through friends

○ Other ____

Q20. In the past 6 months, have you had anal sex with your stable male sex partner(s)?

○ Yes

○ No (Skip to Q23)

Q21. In your last sexual intercourse with your stable male partner, did you or your sex partner use condoms?

○ Yes

○ No

Q22. In your last sexual intercourse with your stable male partner, were your stable male sex partner living with HIV positive?

○ Yes

○ No

○ I do not know

Section 2. Casual male sex partners
*Note: Casual male sex partners mean male sex partners who maintain a sexual relationship with you equal to or less than 3 months, including commercial sex partners (≤3 months).*

Q23. In your lifetime, have you had casual male sex partners?

○ Yes

○ No (Skip to Q30)

Q24. How old were you when you had anal intercourse with your casual male sex partner for the first time?

________years old *(Number input)*

Q25. The first time you had sex with your casual male sex partner, was it consensual?

○ Yes

○ No

*Note: Our definition of consensual is that you were interested in having sex with him, but that you felt like you could have refused or stopped it if you wanted to.*

Q26. Where did you meet your casual male sex partners?

○ Pub, disco, tearoom, or club

○ Spa or bath house, sauna, foot or body massage parlor

○ Park, public restroom, public lawn

○ Website

○ Social media

○ Through friends

○ Other ___

Q27. In the last 6 months, have you had anal sex with your casual sex partner(s)?

○ Yes

○ No (Skip to Q30)

Q28. In your last sexual intercourse with your casual male partner, did you or your sex partner use condoms?

○ Yes

○ No

Q29. In your last sexual intercourse with your casual male partner, have your casual male sex partner living with HIV positive?

○ Yes

○ No

○ I do not know

**Actual risk of HIV infection**

Q30. How many homosexual partners (anal or oral sex) did you have in past 6 months?

○ 1

○ 2-5

○ 6-9

○ ≥10

Q31. Did you have HIV positive homosexual partners in past 6 months?

○ No

○ Have, not know their HIV status

○ Have, all of them had received ART treatment

○ Have, part or all of them had not received ART treatment

Q32. Did you have unprotected anal intercourse with a man in past 6 months?

○ No

○ Sometimes

○ Always

Q33. Did you have commercial sex with a man in past 6 months?

○ Yes

○ No

Q34. Were you diagnosed with sexual transmitted diseases (e.g., syphilis and gonorrhoea) in past 6 months?

○ Yes

○ No

Q35. Did you use recreational drugs (e.g., rush and poppers) in past 6 months?

○ Yes

○ No

Q36. What was your main sex role during homosexual behaviour in past 6 months?

○ Only recipient sex role

○ Recipient or inserted sex role

○ Only inserted sex role

Q37. How often did you have group sex (i.e., have sex with at least two men at the same time) with men in past 6 months?

○ No

○ Sometimes

○ Often

Q38. How likely do you think that men who have sex with men around you have been infected with HIV?

○ No possibility

○ Low possibility

○ High possibility

○ I don’t know

Q39. How likely do you think that you have been infected with HIV in the past 6 months?

○ No possibility

○ Low possibility

○ High possibility

○ I don’t know

Q40. Are you worried about being infected with HIV?

○ Not at all

○ Not worried

○ Worried

○ Very worried

**HIV testing**

Q41. Have you ever been tested for HIV (including both facility-based and self-testing)?

○ Yes

○ No (Skip to Q53)

**Section 1: Facility-based HIV testing**

*The next set of questions will ask about your practices and attitudes in regards to HIV testing (This is facility-based HIV testing, NOT HIV self-testing).*

Q42. Have you ever been tested for HIV in the hospital, CDC or CBO?

○ Yes

○ No (Skip to Q47)

Q43. In the past 6 months, how many times have you had a HIV facility-based testing?

○ 0 (Skip to Q47)

○ 1

○ 2

○ 3

○ 4

○ 5

○ 6

○ >6

Q44. Of these HIV testings in the past 6 months, did you receive post-test counseling?

○ Yes

○ No

Q45. Of these HIV testings in the past 6 months, have you received the positive results?

○ Yes

○ No (Skip to Q47)

Q46. Did you receive a confirmatory Western Blot test?

*Note: Western Blot test means a round of testing to confirm the final result after the initial HIV testing.*

○ Yes

○ No

**Section 2: HIV self-testing**

*The next set of questions will ask about your HIV and STI testing and results. Self-testing refers to you administer the test yourself and interpreting results.*

Q47. Have you ever taken an HIV self-test?

○ Yes

○ No (Skip to Q53)

Q48. In the past 6 months, how many times have you had a HIV self-testing?

○ 0 (skip to Q53)

○ 1

○ 2

○ 3

○ 4

○ 5

○ 6

○ >6

Q49. In the past 6 months, have you ever been administered (given) an HIV self-test from other people?

○ Yes

○ No

Q50. In the past 6 months, did you receive post self-test counseling?

○ Yes

○ No

Q51. In the past 6 months, did you have a positive result in any of your HIV self-test(s)?

○ Yes

○ No (Skip to Q53)

Q52. Did you receive a confirmatory Western Blot test?

*Note: Western Blot test means a round of testing to confirm the final result after the initial HIV testing.*

○ Yes

○ No

**HIV-related knowledge**

Q53. Is AIDS an incurable serious infectious disease?

○ Yes

○ No

○ I don’t know

Q54. Is it possible to be infected with HIV by sharing syringes with people who living with HIV?

○ Yes

○ No

○ I don’t know

Q55. Can we judge whether a person is infected with HIV by his appearance?

○ Yes

○ No

○ I don’t know

Q56. Can the correct use of condoms reduce the risk of infection and transmission of HIV?

○ Yes

○ No

○ I don’t know

Q57. Will the use of new drugs (such as methamphetamine, ecstasy, K-powder, etc.) increase the risk of HIV infection?

○ Yes

○ No

○ I don’t know

Q58. Can participation in methedone maintenance treatment reduce the risk of AIDS infection?

○ Yes

○ No

○ I don’t know

Q59. Should we actively seek HIV testing and counseling services after high-risk behaviors (such as needle sharing drug abuse/unsafe sex)?

○ Yes

○ No

○ I don’t know

Q60. Does the intentional spread of HIV require legal responsibility?

○ Yes

○ No

○ I don’t know

**HIV prevention services**

Q61. Have you ever heard of PrEP (pre-exposure prophylaxis)?

*Note: PrEP (pre-exposure prevention) refers to the method that people who have not been infected with HIV take specific antiviral drugs to prevent HIV infection before they having potential HIV exposure.*

○ Yes

○ No

Q62. Are you willing to use PrEP (pre-exposure prophylaxis) before potential HIV exposure?

○ Yes

○ No

Q63. Have you ever used PrEP (pre-exposure prophylaxis) before?

○ Yes

○ No

Q64. Have you ever heard of PEP (post-exposure prophylaxis)?

*Note: PEP (post-exposure prophylaxis) refers to the method in which individuals who have not yet been infected with HIV take specific antiviral drugs within 72 hours after having high-risk behaviors with HIV infected individuals or those with unknown infection status to prevent HIV infection.*

○ Yes

○ No

Q65. Are you willing to use PEP (post-exposure prophylaxis) after HIV exposure?

○ Yes

○ No

Q66. Have you ever used PEP (post-exposure prophylaxis) before?

○ Yes

○ No

**Social network**

Q67. How many male friends do you have like this: When facing difficulties in life, you are willing to seek help from him or confide in him about sensitive privacy issues

○ 0

○ 1~5

○ 6~9

○ ≥10

Q68. Have your male friend mentioned above been tested for HIV in the past 6 months?

○ None of my friends have been tested

○ Some of my friends have been tested

○ All my friends have been tested

○ I don’t know

Q69. Do you know the HIV infection status of the above male friends?

○ None of my friends have been infected with HIV

○ Some of my friends have been infected with HIV

○ I don’t know

Q70. Have you participated in any activities organized by local community-based organization?

○ I have participated

○ I have not participated

Q71. In the last 6 months, which of the following services did you receive:

○ Condom distribution

○ Lubricant distribution

○ Peer Education

○ STD Diagnosis or Treatment

○ HIV counseling or Testing

○ AIDS/STD Materials (pamphlets, etc.)

○ Medical treatment (methadone, ART, etc.)

○ Needle exchange

○ None of the above

**Social media**

Q72. Have you ever used gay social apps (such as Blued)?

○ Yes

○ No (Skip to Q75)

Q73. How long have you been registering for gay social apps?

○ <1 year

○ ≥1 year

Q74. How often did you use gay social apps?

○ <1 time per week on average

○ 1-6 times a week on average

○ Once a day on average

○ 2-4 times a day on average

○ ≥5 times a day on average

Q75. Have you ever seen anything related to AIDS on Weibo, Wechat, QQ messages or mobile Apps?

○ Yes

○ No

Q76. Have you ever looked online for HIV-related information?

○ Yes

○ No

**Perceived risk of HIV infection**

Q77. Do you aware the severity of HIV prevalence among men who have sex with men in China?

○ Not at all

○ Aware

○ Well aware

Q78. Do you think the HIV prevalence is serious among men who have sex with men in China?

○ Rarely zero

○ Relatively low

○ Mode rate

○ Relatively high

○ Serious

Q79. Do you aware the severity of HIV prevalence among men who have sex with men in your social network?

○ Not at all

○ Aware

○ Well aware

Q80. Do you think the HIV prevalence is serious among men who have sex with men in your social network?

○ Rarely zero

○ Relatively low

○ Mode rate

○ Relatively high

○ Serious

**Ending section**

Please scan the QR code below to add our WeChat named “Health Survey of Shandong University” to get reward. Please note your phone number when you add us. We will not share your personal information to anyone and will not contact you except for the situation of follow up notifications and reward deliveries. After we finish all the follow up surveys, your contact will be deleted.

[QR code]

*PS: Different cities have different QR codes. Which QR code will be showed here decided by the Question 1 (Q1) in Part II. This paragraph is not included in the actual questionnaire.*

Please input our WeChat ID:

_________________________________ *(WeChat number input)*

Thank you very much! You have finished all the questions now.

Please be assured that all your personal information will be protected by a password secured phone. Only staff who would send out the reminder and reward could see your account. We will not share your personal information to anyone and will not contact you except for the situation of follow up notifications and reward deliveries. After we finish all the follow up surveys, your contact will be deleted.
