# Supplementary material for: Associations between perceived and actual risk of HIV infection and HIV prevention services uptake among men who have sex with men in Shandong province, China: a cross-sectional study
Source: BMC Public Health. 2024 Jun 1;24:1470. doi: 10.1186/s12889-024-18985-x (PMC11143659; doi:10.1186/s12889-024-18985-x)
Supplement: Supplementary file 2 — Supplementary Material 2. [file 12889_2024_18985_MOESM2_ESM.docx]

Supplementary file 2. The HIV risk assessment scale.

| Number | Items | Response | Score |
| --- | --- | --- | --- |
| 1 | How many homosexual partners (anal or oral sex) did you have in past 6 months? | 1 | 0 |
|  |  | 2-5 | 1 |
|  |  | 6-9 | 2 |
|  |  | ≥10 | 3 |
| 2 | Did you have HIV positive homosexual partners in past 6 months? | No | 0 |
|  |  | Have, all of them had received ART treatment | 1 |
|  |  | Have, not know their HIV status | 2 |
|  |  | Have, part or all of them had not received ART treatment | 3 |
| 3 | Did you have unprotected anal intercourse with a man in past 6 months? | No | 0 |
|  |  | Sometimes | 1 |
|  |  | Always | 2 |
| 4 | Did you have commercial sex with a man in past 6 months? | No | 0 |
|  |  | Yes | 1 |
| 5 | Were you diagnosed with sexual transmitted diseases (e.g., syphilis and gonorrhoea) in past 6 months? | No | 0 |
|  |  | Yes | 1 |
| 6 | Did you use recreational drugs (e.g., rush and poppers) in past 6 months? | No | 0 |
|  |  | Yes | 1 |
| 7 | What was your main sex role during homosexual behaviour in past 6 months? | Only recipient sex role | 2 |
|  |  | Recipient or inserted sex role | 1 |
|  |  | Only inserted sex role | 0 |
| 8 | How often did you have group sex (i.e., have sex with at least two men at the same time) with men in past 6 months? | No | 0 |
|  |  | Sometimes | 1 |
|  |  | Often | 2 |
