# Supplementary material for: Associations between perceived and actual risk of HIV infection and HIV prevention services uptake among men who have sex with men in Shandong province, China: a cross-sectional study
Source: BMC Public Health. 2024 Jun 1;24:1470. doi: 10.1186/s12889-024-18985-x (PMC11143659; doi:10.1186/s12889-024-18985-x)
Supplement: Supplementary file 3 — Supplementary Material 3. [file 12889_2024_18985_MOESM3_ESM.docx]

Supplementary file 3. Perceived risk of HIV infection among participants (N=1136)

| Perceived risk of HIV infection | n(%) |
| --- | --- |
| HIV risk of infection of themselves |  |
| Not possible | 642(56.6) |
| Low | 464(40.8) |
| High | 29(2.6) |
| HIV prevalence among local MSM population |  |
| Zero | 212(18.7) |
| Relatively low | 409(36.0) |
| Moderate | 300(26.4) |
| Relatively high | 187(16.5) |
| Serious | 28(2.5) |
| HIV prevalence among Chinese MSM population |  |
| Zero | 8(0.7) |
| Relatively low | 138(12.1) |
| Moderate | 343(30.2) |
| Relatively high | 556(48.9) |
| Serious | 91(8.0) |
