# Supplementary material for: Associations between perceived and actual risk of HIV infection and HIV prevention services uptake among men who have sex with men in Shandong province, China: a cross-sectional study
Source: BMC Public Health. 2024 Jun 1;24:1470. doi: 10.1186/s12889-024-18985-x (PMC11143659; doi:10.1186/s12889-024-18985-x)
Supplement: Supplementary file 4 — Supplementary Material 4. [file 12889_2024_18985_MOESM4_ESM.docx]

Supplementary file 4. Actual risk of HIV infection (N=1136)

| HIV risk assessment scale | n(%) |
| --- | --- |
| How many homosexual partners (anal or oral sex) did you have in past 6 months |  |
| 1 | 630(55.5) |
| 2-5 | 462(40.7) |
| 6-9 | 31(2.7) |
| ≥10 | 13(1.1) |
| Did you have HIV positive homosexual partners in past 6 months |  |
| No | 926(81.5) |
| Have, all of them had received ART treatment | 8(0.7) |
| Have, not know their HIV status | 199(17.5) |
| Have, part or all of them had not received ART treatment | 3(0.3) |
| Did you have unprotected anal intercourse with a man in past 6 months |  |
| No | 810(71.3) |
| Sometimes | 284(25.0) |
| Always | 42(3.7) |
| Did you have commercial sex with a man in past 6 months |  |
| No | 1090(96.0) |
| Yes | 46(4.0) |
| Were you diagnosed with sexual transmitted diseases (e.g., syphilis and gonorrhoea) in past 6 months |  |
| No | 1114(98.1) |
| Yes | 22(1.9) |
| Did you use recreational drugs (e.g., rush and poppers) in past 6 months |  |
| No | 985(86.7) |
| Yes | 151(13.3) |
| What was your main sex role during homosexual behaviour in past 6 months |  |
| Only recipient | 410(36.1) |
| Recipient or inserted | 393(34.6) |
| Only inserted | 333(29.3) |
| How often did you have group sex (i.e., have sex with at least two men at the same time) with men in past 6 months |  |
| No | 986(86.8) |
| Sometimes | 148(13.0) |
| Often | 2(0.2) |
