# Supplementary material for: Associations between perceived and actual risk of HIV infection and HIV prevention services uptake among men who have sex with men in Shandong province, China: a cross-sectional study
Source: BMC Public Health. 2024 Jun 1;24:1470. doi: 10.1186/s12889-024-18985-x (PMC11143659; doi:10.1186/s12889-024-18985-x)
Supplement: Supplementary file 5 — Supplementary Material 5. [file 12889_2024_18985_MOESM5_ESM.docx]

Supplementary file 5. The relationships between actual and perceived risk of HIV infection and HIV prevention services uptake (N=1136)

| Variables | HIV testing uptake | | Willingness to use PrEP | | History of PrEP use | | Willingness to use PEP | | History of PEP use | |
| --- | --- | --- | --- | --- | --- | --- | --- | --- | --- | --- |
|  | aOR | post p | aOR | post p | aOR | post p | aOR | post p | aOR | post p |
| Perceived risk of themselves | 1.030 | 0.206 | 1.004 | 0.194 | 4.486 | 0.994 | 0.971 | 0.199 | 3.144 | 0.952 |
| Perceived HIV prevalence among social networks | 1.156 | 0.547 | 1.120 | 0.310 | 1.280 | 0.572 | 0.965 | 0.219 | 1.502 | 0.786 |
| Perceived HIV prevalence among national MSM | 1.067 | 0.409 | 1.903 | 0.943 | 1.038 | 0.232 | 1.737 | 0.829 | 0.986 | 0.230 |
| Actual risk | 0.995 | 0.176 | 0.964 | 0.231 | 1.139 | 0.371 | 1.009 | 0.189 | 1.890 | 0.950 |
| Age | 0.983 | 0.231 | 0.816 | 0.516 | 1.028 | 0.214 | 0.947 | 0.257 | 0.969 | 0.248 |
| Marital status | 1.000 | 0.190 | 0.949 | 0.260 | 1.084 | 0.299 | 1.257 | 0.424 | 1.128 | 0.385 |
| Highest education level | 0.999 | 0.198 | 1.072 | 0.295 | 0.974 | 0.199 | 1.112 | 0.329 | 0.994 | 0.209 |
| Monthly income | 1.198 | 0.718 | 1.027 | 0.224 | 1.014 | 0.181 | 1.175 | 0.410 | 0.898 | 0.404 |
| Gender identity | 1.222 | 0.482 | 1.127 | 0.278 | 0.932 | 0.216 | 0.940 | 0.212 | 0.979 | 0.211 |
| Sexual orientation | 0.966 | 0.274 | 1.112 | 0.356 | 1.040 | 0.222 | 1.031 | 0.211 | 1.083 | 0.322 |
| Disclosure of sexual intercourse with men to others | 0.913 | 0.472 | 1.048 | 0.264 | 1.084 | 0.316 | 1.075 | 0.287 | 1.054 | 0.289 |
| Knowledge about HIV | 1.063 | 0.309 | 1.088 | 0.277 | 0.964 | 0.197 | 1.309 | 0.470 | 1.012 | 0.210 |
| Participated in HIV/AIDS related activities organized by CBOs | 1.281 | 0.856 | 0.991 | 0.198 | 1.050 | 0.260 | 0.949 | 0.259 | 1.131 | 0.440 |
| Ever used MSM social software | 1.146 | 0.399 | 0.973 | 0.208 | 0.960 | 0.189 | 0.857 | 0.268 | 0.922 | 0.256 |
| Ever contacted HIV-related information through apps | 1.364 | 0.778 | 1.302 | 0.490 | 1.138 | 0.307 | 1.893 | 0.772 | 0.991 | 0.206 |
| Ever sought sexual partners through web | 1.306 | 0.630 | 0.649 | 0.463 | 1.017 | 0.172 | 0.966 | 0.206 | 1.074 | 0.252 |

Note: aOR=the logarithmic to the power of the Bayesian model average estimate; post p=posterior probability
